# Assessing the Factors Associated With Iran’s Intra-Industry Trade in Pharmaceuticals

**DOI:** 10.5539/gjhs.v7n5p311

**Published:** 2015-03-30

**Authors:** Hassan Yusefzadeh, Mohammad Hadian, Hassan Abolghasem Gorji, Hossein Ghaderi

**Affiliations:** 1 Department of Health Economics, School of Health Management and Information Sciences, Iran University of Medical Sciences, Tehran, Iran; 2 Department of Health Services Management, School of Health Management and Information Sciences, Health Management and Economics Research Center, Iran University of Medical Sciences, Tehran, Iran

**Keywords:** intra-industry trade, pharmaceutical products, Grubel-Lloyd index, product differentiation

## Abstract

**Background:**

Pharmaceutical industry is a sensitive and profitable industry. If this industry wants to survive, it should be able to compete well in international markets. So, study of Iran’s intra-industry trade (IIT) in pharmaceuticals is essential in order to identify competitiveness potential of country and boost export capability in the global arena.

**Methods:**

This study assessed the factors associated with Iran’s intra-industry trade in pharmaceuticals with the rest of the world during the 2001–2012 periods using seasonal time series data at the four-digit SITC level. The data was collected from Iran’s pharmaceutical Statistics, World Bank and International Trade Center. Finally, we discussed a number of important policy recommendations to increase Iran’s IIT in pharmaceuticals.

**Results:**

The findings indicated that economies of scale, market structure and degree of economic development had a significantly positive impact on Iran’s intra-industry trade in pharmaceuticals and tariff trade barriers were negatively related to IIT. Product differentiation and technological advancement didn’t have the expected signs. In addition, we found that Iran’s IIT in pharmaceuticals have shown an increasing trend during the study period. Thus, the composition of Iran trade in pharmaceuticals has changed from inter-industry trade to intra-industry trade.

**Conclusions:**

In order to get more prepared for integration into the global economy, the development of Iran’s IIT in pharmaceuticals should be given priority. Therefore, paying attention to IIT could have an important role in serving pharmaceutical companies in relation to pharmaceutical trade.

## 1. Introduction

Pharmaceuticals are considered as one of the essential needs of human beings which are produced using high level of technology. For this reason in trade arena, pharmaceuticals have always enjoyed strategic importance and hence governments attempt to pay specific attention to its production and trade along with other basic goods (Bidel & Camousi, 2009).

The rate of profitability, high turnover and employment of expert work force besides creating economic prosperity, engagement and promotion of knowledge power as well as making countries interdependent have given prominence to the pharmaceutical industry (Kebriaeezadeh, Koopaei, Abdollahiasl, Nikfar, & Mohamadi, 2013). The value of world pharmaceutical market with a share of 14 percent of the world GDP was 954 billion dollars in 2012. Average growth in the global pharmaceutical market has been 5.5% in the last decade (International Trade Center, 2012).

Drug costs in Iran and many other developing countries accounts for about 30% of the total health care expenditure. While in most OECD (Organization for Economic Cooperation and Development) member countries, the share of drug costs in the total health care expenses is less than 10 percent (Basmanjy, Izadi, & Behbahani, 2008). Health care level of a country depends mostly on the accessibility of pharmaceuticals, thus, pharmaceutical trade wants more consideration of policy makers. The world pharmaceutical trade has expanded significantly because of world pharmaceutical consumption and production has increased in the recent years (Davari, Walley, & Haycox, 2011). But Iran’s pharmaceuticals constitute a small share of international trade.

Pharmaceutical industry is ranked the second largest industry following electronic industry. Large pharmaceutical companies try to greatly increase their property by spending a lot of money to produce new drugs. In fact, pharmaceutical industry is a sensitive and advantageous industry (Cheraghali, 2010). According to the Iranian food and drug department, the share of Iran’s pharmaceutical industry is about 2.6 billion of the 1200 billion dollars, while the share of Turkey as one of Iran’s neighbors is 6 billion dollars (Pharmaceutical Statistics, 2012).

The countries that have appropriate positions in world’s economy usually undergo insignificant changes when confronted with tension. These countries are paving the way for maintaining more facilitation in their pharmaceutical commerce. They have mainly realized the fact that what brings about survival among international competitors is not only the degree of a country’s self-sufficiency, but also, and more importantly, the extent to which it strengthens its position in international markets (Izadian, 2012).

Because of pharmaceutical industry needs a high level of technological abilities in production, it appears to be a capital-intensive industry (Faegh, 2009). So, intra-industry trade results from production efficiency in large scale, product differentiation and imperfect competition in this section (Lu, 2011). Intra-industry trade helps countries find the real comparative advantages of their pharmaceutical products by comparing themselves with other countries based on production methods and technologies. As we are seeking a suitable model for manufacturing, export and import of pharmaceuticals in order to promote and expand our foreign ties, knowing IIT are of utmost importance (Barouni, Ghaderi, & Banouei, 2012).

Iran has numerous relative advantages regarding the pharmaceutical manufacturing conditions including access to infinite energy resources, relative progress in pharmaceutical technologies, high potential to enter into Islamic countries’ markets, relative experience in pharmaceutical manufacturing, and cheap and young expert work force. These advantages can play an effective and competitive role in the Iran’s IIT (Cheraghali, 2010).

In addition, Iran is increasingly becoming integrated into the global economy. Thus, achieving a successful trade performance in pharmaceutical industry is of utmost importance in Iran’s economy. Paying attention to economic integration through removing barriers of bilateral trade, creating trade zones, customs unions and other preferential trade arrangements will result in implementation of common economic policies in countries that have close economic ties with Iran. So, the share of intra-industry trade and the total trade between Iran and these countries will increase (Nasiri & Asl, 2013).

Furthermore, the desirable participation of pharmaceutical industry in trade and absorbing foreign exchange can introduce this sector as the engine of economic growth. Regarding the importance of intra-industry trade in promoting of non-oil exports, it is recommended to take the determinants of intra-industry trade into consideration while protecting and maintain the relative comparative advantages. Moreover, given the low level of Iran’s IIT of pharmaceuticals, trade liberalization is likely to have significant adjustment costs that can be reduced by promoting IIT of this industry (Shabaninejad, Mehralian, Rashidian, Baratimarnani, & Rasekh, 2014). Therefore, study of the factors associated with Iran’s intra-industry trade (IIT) in pharmaceutical industry is necessary in order to identify competitive potential of country and enhance export ability in the global arena and can be considered in foreign trade planning as a strategic and decision-making tool.

In Iran, no study related with IIT of pharmaceutical industry was found. Though, there are many studies associated to different economic sectors some of which would be explained.

The study in China has been performed by Zhang et al. (2005) named Chinese Bilateral Intra-Industry Trade: A Panel Data Study for 50 Countries in the 1992–2001 periods. In this study, five intra-industry trade drivers including Foreign Direct Investment (FDI), geographical distance, economic size, trade openness and trade composition has been considered. They observed that these factors had significant influences on IIT (Zhang, van Witteloostuijn, & Zhou, 2005). The other study associated with the determinants of IIT has been carried by Kabir, et al. (2010) in the 1995-2008 periods by Gravity model. They found that trade liberalization, economic size and FDI had positive effects on IIT, but geographical distance had negative impact (Kabir & Salim, 2010). Caetano and Galego (2006) examined the extent and pattern of intra-industry trade among Central and Eastern European Countries and the European Union during the time 1993 to 2001 using the Grubel-Lloyd index. They found a significant increase in intra-industry trade due to positive effects of economic size, Foreign Direct Investment (FDI), and human capital but negative impacts of geographical distance (Caetano & Galego, 2006). Similar results were obtained by Egger (2002), over the 1985 to 1996, who demonstrated that the market size and distance had positive and negative impact on IIT of OECD manufacturing, respectively (Egger, 2002). Rasekhi (2008) examined intra industry trade (IIT) of Iran’s agricultural products by indices of Grubel & Lioyd and Fontagn & Freudenberg during time period 1997-2003. The Results showed small but rising IIT for agricultural products of Iran. Particularly, the IIT of Iran’s agricultural products increased from 2.73 in 1997 to 5.98 percent in 2003, reflecting comparative advantage have an important role in foreign trade of agricultural products in Iran (Rasekhi, 2008). The study of Turkcan (2011) found IIT between Turkey and other selected OECD countries for the period 1985 to 2000 was negatively related to distance in both final products and intermediate products (Türkcan, 2011). Investigation by Sawyer, et al. (2010) showed that IIT was positively related to income per capita of its trading partners. Also, removal of trade barriers had a positive impact on trade intensity (Sawyer, Sprinkle, & Tochkov, 2010).

## 2. Methods

**Theory of Intra-Industry Trade**

Globalization of economy is the most important and transparent distinctive feature of today’s economy. This means that borders are being opened, trade is promoted, and regional trade and economic unions and zones are expanded. Various criteria are used to measure and determine a country’s trade potential and competitiveness. Intra-industry trade is a suitable criterion that can show the trade flows between the economic sectors of two countries and their trade relations. The common theories of international trade only consider the inter-industry trade (trade of different products associated with different industries) and use simple assumptions such as the existence of a perfect competitive market, homogeneity of goods and increasing returns to scale. Thus, a major share of trade that is associated with the simultaneous trade of goods in a certain industry remains unexplained. Therefore, in the late 1970s, the intra-industry trade theories gradually started to develop and become widespread. Such theories are based on assumptions including increasing returns to scale and imperfect competition (Rasekhi, 2008).

Intra-industry trade is an empirical phenomenon that has been interred into international trade discussions in recent years. Intra-industry trade takes place when a country simultaneously exports and imports of goods or services which produced in the same industry. If goods are differentiated by their appearance or horizontal distinctions, we are dealing with what is known as horizontal intra-industry trade. If goods are differentiated by quality and numerical distinctions, the phenomenon is called vertical intra-industry trade (Gullstrand, 2002). This type of trade is distinct from inter-industry trade. In inter-industry trade, a country is specialized in the production and export of a certain good or service and exports it instead of a different product or service in which it does not have any comparative advantage. The intra-industry trade phenomenon is a very important issue in developing countries. Paying attention to this phenomenon helps countries become aware of their own trade potentials and enhance their trade ties with each other and other countries (Bano, 2002).

In summary, intra-industry trade is as a consequence of variation in consumer preferences and economies of scale which occurs under monopolistic or imperfect competition (Blanes & Martín, 2000).

**Measurement of Iran’s intra-industry trade in pharmaceuticals**

The most commonly used measure of IIT proposed by Grubel and Lloyd. In this index, inter-industry trade is calculated as the ratio of the absolute difference between exports and imports (net trade) over the total trade of the industry. In other words, intra-industry trade is expressed as the ratio of the total trade remaining after subtraction of net trade over the total trade of the industry. In summary, the Grubel-Lloyd Index calculates intra-industry trade as the difference between inter-industry trade and one.


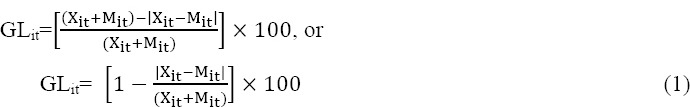


Where X_it_ and M_it_ denote the values of export and import of industry i in year t, respectively. The index includes bounded interval of zero and 100. The index of Grubel-Lloyd (GL_i_) equals 100 if all trade is intra-industry trade (i.e. imports and exports of country from the same industry are equal) and if the extent of GL_i_ is zero, the total trade is inter- industry trade and there is no intra-industry trade in that industry (Wei, 2004).

**Major associated hypothesises (variables) with Iran’s intra-industry trade in pharmaceuticals with the rest of the world**

On the basis of advanced theories of trade and previous empirical studies about intra-industry trade, the following elements used as explanatory variables in this analysis to measure Iran’s intra-industry trade in pharmaceuticals with the rest of the world, these include: (1) product differentiation (PDIF_i_); (2) economies of scale (ES_i_); (3) Market Structure (MS_i_); (4) degree of economic development (DD_i_); (5) technological advancement (KL_i_) and (6) trade barriers (TR_i_). These factors are vitally important in the explanation of IIT in pharmaceutical industry that is chosen for in the analyses.

**Hypothesis 1: a direct relationship exists between product differentiation and Iran’s IIT in pharmaceuticals.**

Pharmaceutical productions are research-oriented activities; product differentiation in pharmaceuticals (by style, quality or technology) will be measured by the ratio of R&D expenditures (RDX_i_) to the value added (VA_i_) in Iran’s pharmaceutical products. If different countries have high technology in different sections of the same industry, the ratio has a positive impact on IIT, and if one country has a higher degree of technology in production than the others, the ratio of R&D expenditure to value added has a negative impact on IIT (Rice, Stewart, & Venables, 2002).


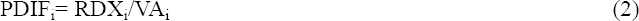


It is assumed that an increase in research and development costs relative to value added will increase the versions of technologically differentiated pharmaceutical products. Thus, it will have a positive effect on the extent of intra-industry trade.

**Hypothesis 2: it is expected that a positive relationship exists among economies of scale and Iran’s IIT in pharmaceuticals.**

Since large economies can readily gain from economies of scale, they can make use of benefits resulting from their large domestic market easily and so, have higher extent of IIT (Sharma, 2000). The ratio of pharmaceutical turnover (total sales) (TUR_i_) to the number of pharmaceutical companies (MS_i_) are used to compute economies of scale (ES_i_) for Iran’s pharmaceutical industry, in this research.


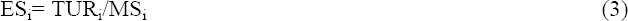


It is assumed that a positive relationship exists between economies of scale and intra-industry trade in pharmaceuticals.

**Hypothesis 3: Market structure is positively related to the level of Iran’s IIT in pharmaceuticals.**

In theory, industries with a large number of companies manufacturing differentiated products are more likely to engage in a higher level of bilateral trade rather than those with only a few companies. The variety of differentiated pharmaceutical products will increase under imperfect competition when a large number of pharmaceutical companies enter the market (Greenaway, Hine, & Milner, 1995). It is expected that the number of pharmaceutical companies (MS_i_) have a positive effect on the level of intra-industry trade.

**Hypothesis 4: The relationship among Iran’s IIT in pharmaceuticals and the degree of economic development is positive.**

A high per capita GNP indicates that the income level is high. As per capita income grows, consumers will demand more of differentiated products in their bilateral trade (Ekanayake, 2001). In this study, Iran’s per capita GNP is applied as a measure of the degree of Iran’s economic development (DD_i_) that would be expected to have a positive relationship with Iran’s IIT in pharmaceuticals.

**Hypothesis 5: Iran’s IIT in pharmaceuticals have a positive relationship with technological advancement.**

The higher ratio of capital relative to labor force in a country result in more likely an increase in the production of differentiated products and increasingly engage in IIT (Helpman, 1981). Pharmaceutical production in a country also depends on the level of capital formation. The ratio of total capital to the labor force (KL_i_) is used in this study to demonstrate Iran’s technological advancement.


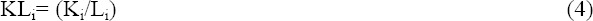


Where, K_i_ and L_i_ are the real capital formation and the total labor force of Iran. This ratio is assumed to have a positive relationship with IIT.

**Hypothesis 6: Trade barriers have a negative effect on Iran’s IIT in pharmaceuticals.**

Eliminating trade barriers facilitates the development of IIT and causes countries can enter each other’s markets with less effort. Imposing trade restrictions such as tariff and non-tariff trade barriers in a particular industry help to encourage domestic manufacturing of the industry, but decrease the volume and range of traded pharmaceutical products (Tan & Cai, 2010). Tariff rate is used in this study to measure the trade barriers for pharmaceutical products. It is assumed that TR_i_ is negatively associated with Iran’s intra-industry trade in pharmaceuticals.

In the next step of the analysis, the log-linear and log-log models are estimated and tested using diagnostic statistics to find a suitable model in order to investigate the impact of six independent variables on the Iran’s IIT in pharmaceuticals.


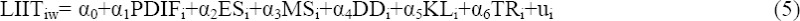


The logarithmic conversion of the Grubel-Lloyd index is calculated using the following function:


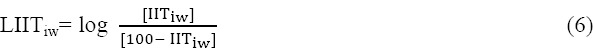


It is expected that α_0_, α_1_, α_2_, α_3_, α_4_ and α_5_ will be positive, and α_6_ will be negative.

The data used in the estimation of Iran’s IIT in pharmaceuticals with the rest of the world were obtained from Iran’s Ministry of Industry, Mine and Trade, Iran’s pharmaceutical Statistics, the Islamic Republic of Iran’s Customs Administration, and World Bank and International Trade Center. The seasonal time series for the period 2001-2012 were processed and coded according to a 4-digit level based on the International Classification’s Harmonized System (3001 to 3006). Data were available in annual format, for increasing the number of observations we converted them to seasonal format. Data consisted of 48 observations for each variable. Also, the analysis was conducted on industry level, not at firm level.

## 3. Results

The results presented in Table 1 show that Iran’s intra-industry trade in pharmaceuticals with the rest of the world has increased from 5.97 in 2001 to 19.85 in 2012. As a result, the composition of Iran trade in pharmaceuticals has changed from inter-industry trade to intra-industry trade. Increasing the number of produced pharmaceuticals and improving product differentiation in this sector appear to be important reasons of the increase in the extent of Iran’s intra-industry trade in pharmaceuticals. As a result, the rate of export growth has increased in the industry over the period. However, the level of Iran’s intra industry trade in pharmaceuticals relative to other major exporting countries was small.

**Table 1:** Comparison of Grubel-Lloyd index of intra-industry trade in pharmaceuticals for Iran and other major exporting countries with the rest of the world, selected years during 2001-2012

Country	2001	2006	2012
Iran	5.97	10.22	19.85
Germany	69.92	86.60	77.70
Belgium	91.18	94.95	87.37
France	74.96	80.86	86.31
Italy	96.42	98.64	98.71
Japan	63.12	49.23	25.67
Switzerland	70.97	66.50	54.56
U.K.	82.49	81.31	89.62
U.S.	87.61	74.42	76.71

Based on results of table 1, Italy, United Kingdom, Belgium, France, Germany and United States had high intra-industry trade indexes and were about net exporters of pharmaceuticals. On the contrary, the intra-industry trade index was partly low for Switzerland. Switzerland has the maximum revealed comparative advantage than all pharmaceutical exporting countries; therefore, Switzerland’s IIT with the rest of the world was low since the pharmaceutical exports were much greater than its imports.

The results of diagnostic tests showed that for the model of Iran’s intra-industry trade in pharmaceuticals with the rest of the world, the log-linear form was appropriate. Furthermore, Augmented Dickey-Fuller (ADF) test were used to test for the presence of unit root in each series of data. Due to non stationary of variables, we had to test for cointegration. The Engle-Granger test confirmed that the Long run relationships between the variables. The estimated coefficients of the function for Iran’s intra-industry trade in pharmaceuticals with the rest of the world along with the expected signs of the coefficients of the explanatory variables are represented in Table 2.

**Table 2:** Iran’s intra-industry trade in pharmaceuticals with the rest of the world

Explanatory variable	expected sign	coefficient	t-statistics	probability
Intercept (C)	-1.340851	-5.565277	0
Product differentiation (PDIF_i_)	+	-9.279581	-1.207831	0.2342
Economies of scale (ES_i_)	+	0.008618	2.420881	0.0201
Market structure (MS_i_)	+	0.018031	4.287388	0.0001
Degree of economic development (DD_i_)	+	0.000173	1.704441	0.0961
Technological advancement (KL_i_)	+	-0.177957	-2.829367	0.0073
Trade barriers (TR_i_)	-	-0.008936	-4.136412	0.0002
R-squared	0.98062			
Adjusted R-squared	0.977142			
Durbin-Watson stat	2.2			
F-statistic	115.2			

As shown in Table 1, economies of scale (ES_i_), market structure (MS_i_), degree of economic development (DD_i_) and trade barriers (TR_i_) had statistically significant impacts on Iran’s intra-industry trade in pharmaceuticals. The coefficients of product differentiation (PDIF_i_) and technological advancement (KL_i_) didn’t have the expected signs; however, technological advancement variable unlike product differentiation variable was statistically significant.

As expected, economies of scale in Iran’s pharmaceutical industry had a significantly positive effect on the extent of intra-industry trade. Market structure was positively related to Iran’s intra-industry trade in pharmaceuticals with the rest of the world, which means an increase in the number of pharmaceutical companies will result in a higher extent of intra-industry trade. The coefficient of DD_i_ was positive and significant at the 10 percent level. These results confirm that the degree of economic development has a positive influence on intra-industry trade. Therefore, an increase in income will cause a large demand for pharmaceutical products. The coefficient of TR_i_ was negative and significant, indicating that tariff trade barriers have a negative impact on the extent of Iran’s intra-industry trade in pharmaceuticals.

Contrary to expectations, the effect of product differentiation (PDIF_i_) on Iran’s intra-industry trade in pharmaceuticals was negative. Applying the ratio of R&D expenditures to value added as a criterion for product differentiation may have a negative impact on IIT in countries with a high technological advancement in that industry. Thus, Iran may have a partial technological strength in pharmaceutical production which causes this country to participate in low extent of intra-industry trade in pharmaceuticals than other exporting countries. This result was consistent with the finding of Karn.

During the study period, the ratio of capital-labor in Iran relative to the level of intra-industry trade in pharmaceuticals increased gradually. However, the level of technological advancement (KL_i_) which was calculated by the ratio of total capital to the labor force had a significantly negative influence on Iran’s IIT in pharmaceuticals with the rest of the world. Therefore, due to the estimated coefficient for KL_i_ was negative, the impact of capital-labor ratio on Iran’s IIT in pharmaceuticals wasn’t supported by the model.

So, a one percent increase in the variables of product differentiation, economies of scale, market structure, degree of economic development, technological advancement and trade barriers will result in -9.27, 0.0086, 0.018, 0.00017, -0.17 and -0.0089 percent rise in Iran’s intra-industry trade with the rest of the world, respectively. In this paper, when product differentiation and technological advancement rise, the extent of intra-industry trade will decline that are not consistent with theoretical expectations.

So far, one single study has been carried out to examine intra-industry trade in pharmaceuticals which has been performed by Karn in Australia. He has studied the models and factors affecting Australia’s international trade in pharmaceutical sector. His research’s results are largely similar to our research. According to Karn’s findings, the variables of economies of scale, market structure and the degree of economic development had a significantly positive impact on Australia’s IIT in pharmaceuticals, but trade barriers had a significantly negative influence (Chuankamnerdkarn, 1997).

## 4. Discussion

Considering extensive literature review, we found 6 factors affecting the IIT in pharmaceuticals with the rest of the world. Overall, three variables affected IIT in pharmaceuticals in the same way. One was economies of scale, which had a significant and positive impact on IIT. Another factor was the influence of market structure. IIT was positively affected by market structure. The last factor was the degree of economic development in terms of per capita GNP. IIT is more likely to take place with large economies than with small ones. Tariff trade barriers had a significantly negative influence. These observations were in line with most theoretical predictions and empirical findings in the intra-industry trade literature. The estimated coefficients for product differentiation and the level of technological advancement of the country didn’t have the expected positive signs, but the variable of the level of technological advancement was significant. Product differentiation had a negative impact on Iran’s intra-industry trade and indicates that Iran may have technological intensity in pharmaceutical production leading to the country’s engagement in less intra-industry trade in pharmaceuticals than other major exporting countries. Therefore, product differentiation is not necessary to increase IIT in every case, but it depends on the situation in which the index is applied. Nevertheless the steady increase in the capital-labor ratio in Iran relative to the extent of intra-industry trade in pharmaceuticals, the influence of the ratio of total capital to the labor force on Iran’s intra-industry trade in pharmaceuticals was negative during the study period.

Also, Iran’s IIT in pharmaceuticals have shown an increasing trend during the period 2001 to 2012. This growth may be due to an increase in the number of produced pharmaceuticals and product differentiation in this sector. The great intra-industry trade indexes for industrial countries seem to be associated with customs unions, high levels of income, common borders, trade liberalization and attainment to larger economies of scale in pharmaceutical production. Low levels of Iran’s Intra-industry trade indicate that Iran is to some extent a net importer of pharmaceuticals.

Due to the low extent of Iran’s intra-industry trade in pharmaceuticals, it seems that there is a considerable potential for developing and promoting intra-industry trade and exports in pharmaceutical industry. However, preparation for integration into the global economy in commodity groups having intra-industry trade is relatively high. Therefore, it seems that in order to get more prepared for integration into the global economy, Iran should pay special attention to promoting intra-industry trade in pharmaceuticals besides protecting the existing comparative advantages.

In order to increase Iran’s IIT in pharmaceuticals, the most important recommendations are as follows:

1). New sources particularly product differentiation and economies of scale are important in establishing a sustainable advantage in pharmaceutical industry. Accordingly improving product differentiation, investing in skilled manpower, creating and extending research and development departments (R&D), and diversifying exports are essential. In addition, improving the level of technology, reducing costs and taking advantage of economies of scale are significant and can be taken into consideration.
2). Country-specific characteristics, such as foreign direct investment and active participation in economic integrations lead to the promotion of intra-industry trade in pharmaceutical industry. Hence, paying serious attention to these factors can result in an increase in pharmaceutical’s intra-industry trade.
3). Development and promotion of intra-industry trade reduce pharmaceutical industry’s demand for support. Therefore, since development of intra-industry trade involves both exports and imports, the pressure from support-seeking parties is reduced and a transparent, strong and permanent strategy can be developed.
4). Due to the chronic shortage of foreign exchange resources in Iran, attracting more foreign investment in pharmaceutical industry is of utmost importance.
5). It is necessary to reduce dependence on imported pharmaceutical primary materials in order to cope with the sharp increase in imports and maintain trade balance. Since pharmaceutical imports are mostly in the form of pharmaceutical primary materials, one of the most important strategies for increasing the competitiveness potential of Iran’s pharmaceutical sector is to reduce import and achieve independence.
6). Developing a transparent and appropriate strategy for constant export of pharmaceutical products is essential for preventing instability in export and market loss.

The major limitations of this study was inaccessibility to statistics of counterfeit and smuggling drugs in Iran’s pharmaceutical market that are considered as a threat to peoples’ health and life.

## 5. Conclusions

In summary, it is concluded that compared with other major exporting countries, the extent of Iran’s intra-industry trade in pharmaceuticals was low. However, Iran’s intra-industry trade in pharmaceuticals with the rest of the world was affected by industry characteristics such as economies of scale, market structure and so on. Given the low level of intra-industry trade in pharmaceutical products, the pharmaceutical competitiveness and globalization rates of Iran are small. So, in order to get ready for joining the global economy, Iran should consider the development of IIT in pharmaceuticals. Therefore, paying attention to intra-industry trade could have a valuable role in helping pharmaceutical companies and policy makers to promote pharmaceutical trade. Moreover, this work partly helps to clarify the path of Iran’s pharmaceutical trade policy.
